# Pan-cancer analysis of bi-allelic alterations in homologous recombination DNA repair genes

**DOI:** 10.1038/s41467-017-00921-w

**Published:** 2017-10-11

**Authors:** Nadeem Riaz, Pedro Blecua, Raymond S. Lim, Ronglai Shen, Daniel S. Higginson, Nils Weinhold, Larry Norton, Britta Weigelt, Simon N. Powell, Jorge S. Reis-Filho

**Affiliations:** 1 Department of Radiation Oncology, Memorial Sloan Kettering Cancer Center, New York, NY 10065 USA; 2 Department of Pathology, Memorial Sloan Kettering Cancer Center, New York, NY 10065 USA; 3 Human Oncology and Pathogenesis Program, Memorial Sloan Kettering Cancer Center, New York, NY 10065 USA; 4 Department of Epidemiology and Biostatistics, Memorial Sloan Kettering Cancer Center, New York, NY 10065 USA; 5 Department of Medicine, Memorial Sloan Kettering Cancer Center, New York, NY 10065 USA

## Abstract

*BRCA1* and *BRCA2* are involved in homologous recombination (HR) DNA repair and are germ-line cancer pre-disposition genes that result in a syndrome of hereditary breast and ovarian cancer (HBOC). Whether germ-line or somatic alterations in these genes or other members of the HR pathway and if mono- or bi-allelic alterations of HR-related genes have a phenotypic impact on other cancers remains to be fully elucidated. Here, we perform a pan-cancer analysis of The Cancer Genome Atlas (TCGA) data set and observe that bi-allelic pathogenic alterations in homologous recombination (HR) DNA repair-related genes are prevalent across many malignancies. These bi-allelic alterations often associate with genomic features of HR deficiency. Further, in ovarian, breast and prostate cancers, bi-allelic alterations are mutually exclusive of each other. The combination of these two properties facilitates reclassification of variants of unknown significance affecting DNA repair genes, and may help personalize HR directed therapies in the clinic.

## Abstract (web summary)

Germline mutations in homologous recombination (HR) DNA repair genes are linked to breast and ovarian cancer. Here, the authors show that mutually exclusive bi-allelic inactivation of HR genes are present in other cancer types and associated with genomic features of HR deficiency, expanding the potential use of HR-directed therapies.

## Introduction

Germline mutations in *BRCA1/BRCA2* are associated with an increased risk of breast, ovarian, prostate and/or pancreatic cancer^1–3^. Pathogenic mutations in these genes not only predispose to cancer, but can also be exploited therapeutically with homologous recombination (HR) directed therapies, such as PARP inhibitors and platinum salts^4, 5^. Genetic alterations in *BRCA1/2* and other HR-related genes are not solely limited to germ-line pre-disposition syndromes; for example, ovarian and advanced prostate cancers not uncommonly display somatic alterations affecting these genes^6, 7^.

Hereditary cancers associated with germ-line mutations in *BRCA1/2* have recently been found to display specific patterns of genomic alterations that likely result from defective HR DNA repair. Certain types of structural abnormalities, such as patterns of loss-of-heterozygosity in long segments (e.g., large-scale state transitions (LST)), have been shown to be associated with mutations in *BRCA1/*2 and may be employed as surrogates for sensitivity to HR-directed therapies^8–11^. *BRCA1/2*-deficient tumors also display a characteristic mutational signature (signature 3), which likely stems from a lack of competent HR DNA repair of double strand breaks^12, 13^. In fact, defects in other DNA repair pathways have also recently been found to leave characteristic marks on the tumor genome, suggesting that genomic features of deficiency in a DNA repair pathway can be identified from whole-exome and whole-genome sequencing data^14, 15^.

Here, we performed a pan-cancer analysis of The Cancer Genome Atlas (TCGA) data set and observed that over 5% of all tumors have a bi-allelic pathogenic alteration in an HR-related gene. We further find that these bi-allelic alterations are significantly associated with genomic features of HR deficiency as described above, whereas mono-allelic alterations are not. Finally, using these cardinal genomic features of HR deficiency and the observation that bi-allelic alterations affecting HR-related genes are mutually exclusive, we were able to facilitate reclassification of some somatic missense mutations in these genes as putatively pathogenic.

## Results

### Bi-allelic alterations in HR genes occur across cancer types

Given that alterations affecting HR-related genes in ovarian, breast, and prostate cancer have been associated with response to HR directed therapies^16, 17^, we sought to define the prevalence and biological impact of pathogenic somatic and germ-line mutations in 102 HR-related genes (i.e., genes curated from the literature as being directly or indirectly related to HR DNA repair^2, 4, 5^; Supplementary Data 1; methods) in 8178 tumors from TCGA. We found that 13% of tumors have at least 1 pathogenic (methods) mono-allelic mutation in one of these genes (Supplementary Fig. 1a, b; Supplementary Datas 2 and 3). *POLQ* was associated with highest number of mono-allelic pathogenic mutations, with a single-nucleotide polymorphism (SNP) lle1421fs displaying a prevalence of 0.6% in the cohort; however, this SNP was not enriched in TCGA when compared with a cohort of patients from exome aggregation consortium^18^. The biological and clinical significance of these *POLQ* alterations remains to be determined. Further studies are warranted to define the functional impact of mono and bi-allelic alterations of *POLQ* on HR DNA repair in human cancers.

**Fig. 1:**
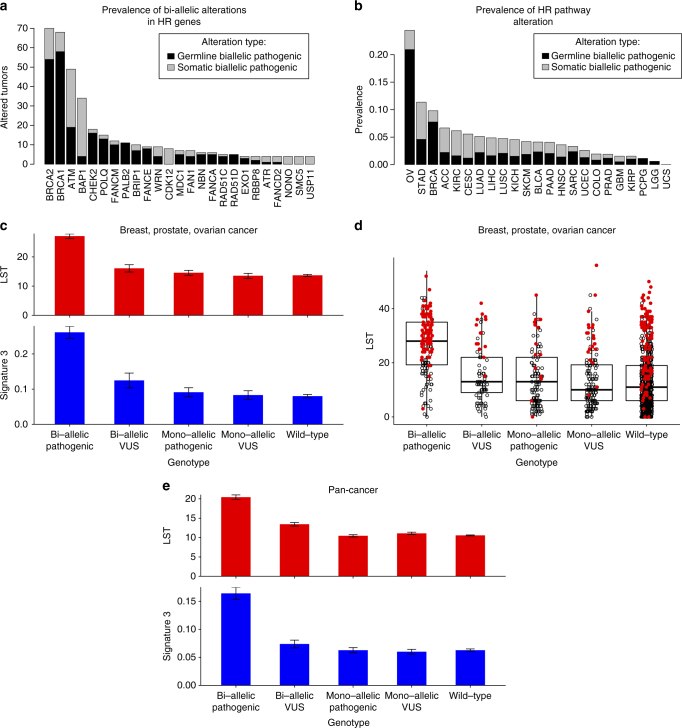
Bi-allelic pathogenic alterations affecting HR-related genes affect multiple cancer types and are associated with genomics features of HR deficiency. **a** Incidence of bi-allelic pathogenic alterations in HR-related genes in TCGA samples stratified according germ-line or somatic origin. It is important to note that germ-line bi-allelic alterations most commonly consisted of a germ-line pathogenic mutation and a somatic loss of heterozygosity. **b** Incidence of bi-allelic alterations of HR-related genes according to cancer type. **c** Association of LST and signature 3 in HBOC cancers. Bi-allelic pathogenic alterations and bi-allelic VUSs in HR-related genes are associated with elevated LST and signature 3 (pathogenic: *p* < 2.2*10^−16^ and *p* < 2.2*10^−16^, respectively; VUS: *p* = 0.02 and *p* = 0.017, respectively). Mono-allelic pathogenic alterations were not associated with either LST or signature 3 (*p* = 0.26 and *p* = 0.14, respectively). **d** LST for each genotype; cases with dominant signature 3 colored red (see methods). The *box plot center line* represents the median, the box limits represent the 1st and 3rd quartiles, respectively, and the *whiskers* extend from box limits to the largest value up to 1.5 times the interquartile range. **e** Pan-cancer analysis shows bi-allelic pathogenic alterations associated with an elevated LST and signature 3 (*p* < 2.2*10^−16^ and *p* < 2.2*10^−16^, respectively). Mono-allelic pathogenic alterations were not associated with elevated LST or signature 3 (*p* = 0.98 and *p* = 0.76, respectively). All *p*-values from the Wilcoxon-rank sum test. *Error bars* represent s.e.m.

As HR-related genes generally act as tumor suppressors and in vitro studies indicate that a complete loss-of-function is required for loss of competent HR DNA repair, we focused our subsequent analyses on bi-allelic pathogenic alterations^19, 20^. As expected, *BRCA1/BRCA2* were the most commonly altered genes, followed by several genes previously associated with cancer predisposition, including *CHEK2, PALB2, RAD51C*, and *RAD51D*(Fig. 1a)^21^. Some HR-related genes were preferentially affected by germline alterations (e.g., *BRCA1/2, CHEK2, FANCM, PALB2*), whereas others (e.g., *ATM, BAP1, CDK12*) were preferentially affected by somatic event (Fig. 1a, Supplementary Fig. 1c, Supplementary Data 2). Of note, germ-line bi-allelic alterations most commonly consisted of a germ-line pathogenic mutation followed by a somatic loss of heterozygosity event (Supplementary Fig. 1c).

Over 5% of all cancers have a bi-allelic pathogenic alteration in HR-related genes, with 25% and 10% of ovarian and breast cancers, respectively, having this pathway altered (Fig. 1b). Across all cancer types, only 45% of bi-allelic alterations in HR-related genes occurred in traditional *BRCA1/*2-associated hereditary cancers (hereditary breast and ovarian cancer (HBOC), namely breast, ovarian, and prostate cancer), suggesting that this pathway is altered in other malignancies (Fig. 1b, Supplementary Fig. 1d, Supplementary Data 3). In gastric, kidney, lung, and bladder cancers, ≥5% of cases were found to be affected. Interestingly, gastric cancers have been previously implicated to have evidence of HR deficiency by analysis of mutational signatures^22^. The nature (i.e., somatic + germline vs. somatic) of bi-allelic hits varied according to anatomical site (Fig. 1b).

### Bi-allelic alterations associate with HR deficiency

**Fig. 2:**
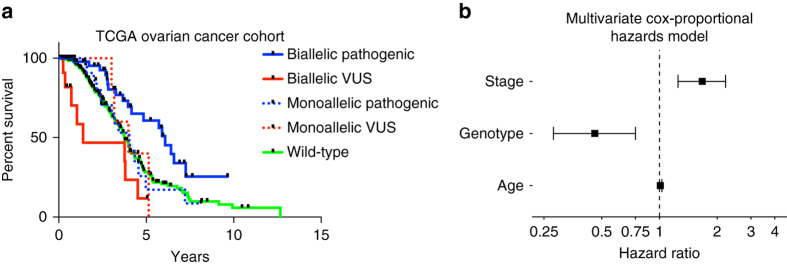
Overall survival (OS) in TCGA ovarian cancer patients who received platinum-based therapy. **a** Bi-allelic alterations in HR-related genes are associated with significantly longer overall survival (OS, *p* = 0.005; log-rank test). **b** Forest plot showing results from a multivariate Cox proportional-hazards model adjusting for stage (continuous: I, II, III, IV) and age (continuous) demonstrates that bi-allelic pathogenic alterations are associated with significantly longer overall survival (hazard ratio (HR) = 0.46, 95% confidence interval (CI) = 0.28–0.75, *p* = 0.002, Genotype is a categorical variable, bi-allelic vs. other). Stage (HR = 1.67, 95% CI = 1.25–2.21, *p* < 0.001) and age (HR = 1.01, 95% CI 1.00–1.03, *p* = 0.02) are also associated with outcome on multivariate analysis

We hypothesized that bi-allelic alterations, but not the majority of mono-allelic alterations, in HR-related genes would result in features consistent with HR DNA repair defects. We first examined if bi-allelic alterations were associated with phenotypic consequences in HBOC cancers. Consistent with our hypothesis, we found that cases with bi-allelic pathogenic alterations had significantly higher LST scores (*p* < 2.2*10^−16^; Wilcoxon-rank sum test) and mutations consistent with signature 3 (*p* < 2.2*10^−16^; Wilcoxon-rank sum test) than wild-type cases, whereas mono-allelic pathogenic alterations did not (Fig. 1c, d). Cases with bi-allelic hits where one of the mutations was a variant of unknown significance (VUS, methods) were also found to display features of HR DNA repair deficiency (Fig. 1c, d). Consistent with the notion that bi-allelic pathogenic alterations may be causative of this phenotype, these cases displayed (i) a significant association with signature 3 (*p* = 2.2*10^–16^; Wilcoxon-rank sum test), (ii) an inverse correlation with the fraction of mutations ascribed to aging-related signatures and the frequency of aging-related signatures as the dominant signature (*p* = 3.0*10^−13^ and *p* = 0.028; for germ-line and somatic mutations, respectively; Fisher’s exact test) (Supplementary Fig. 2), and (iii) with longer overall survival (OS) in a cohort of ovarian cancer patients who uniformly received an HR-directed therapy (platinum-based treatment, *p* = 0.005; log-rank test, Fig. 2). An identical analysis of the pan-cancer data set confirmed that bi-allelic pathogenic alterations affecting HR-related genes were significantly associated with LST (*p* < 2.2*10^−16^;Wilcoxon-rank sum test) and signature 3 (*p* < 2.2*10^−16^;Wilcoxon-rank sum test), whereas mono-allelic alterations were not (*p* = 0.26 and *p* = 0.14 for LST and signature 3 respectively (NS); Wilcoxon-rank sum test, Fig. 1e). These associations remained significant after exclusion of HBOC cancers from the data set (LST *p* = 2.79*10^−16^ and signature 3 *p* = 7.9*10^−4^; Wilcoxon-rank sum test, Supplementary Fig. 3a) and were individually statistically significant in 8 of 12 cancers with at least 10 bi-allelic alterations (LST *p*-values ovarian cancer (OV; *p* = 8.9*10^−11^), breast cancer (BRCA; *p* = 5.4*10^−17^), lung adenocarcinoma (LUAD; *p* = 1.6*10^−3^), lung squamous cell carcinoma (LUSC; *p* = 0.02), SKCM (*p* = 0.04), head and neck squamous cell carcinoma (HNSC; (*p* = 0.02), bladder cancer (BLCA; *p* = 7.8*10^−3^), UCEC (*p* = 0.03)) (Supplementary Fig. 3b), indicating that bi-allelic alterations in HR-related genes likely result in HR DNA repair deficiency regardless of the anatomical site of origin of the cancer. Not only the prevalence of bi-allelic inactivation of HR-related genes but also the nature of these alterations (i.e., germ-line vs. somatic) vary across cancer types (Supplementary Fig. 3c), and impact on the strength of the association between bi-allelic inactivation of HR-related genes and genomic features of HR DNA repair defects in distinct cancer types (Supplementary Fig. 3b, c). In fact, the magnitude of phenotypic influence of a bi-allelic alteration is more conspicuous in cancers where one of the alterations is germ-line than in those where both are somatic, potentially due to the chronology of the genetic events and/or variations in the cell of origin.

### Bi-allelic alterations in HR genes are mutually exclusive

**Fig. 3:**
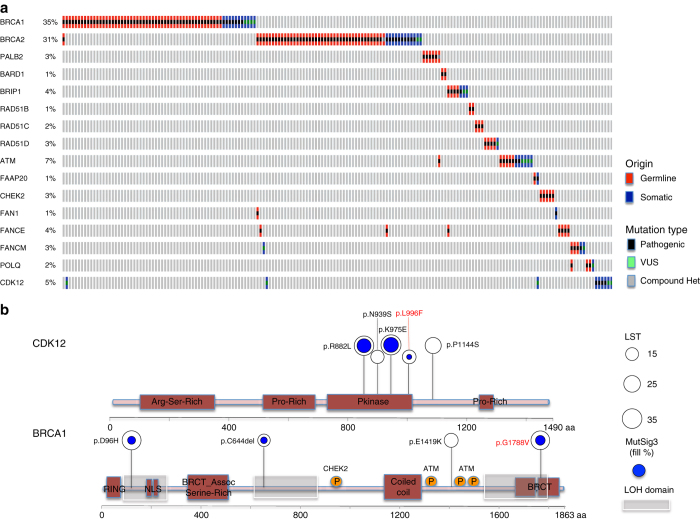
Mutual exclusivity analysis of bi-allelic alterations in HR-related genes and reclassification of VUSs based on genomics information. **a** Bi-allelic pathogenic alterations are mutually exclusive using two different statistical tests (*p* = 0.022, WExT, saddle point approximation of COMET; *p* = 0.025, permutation, methods). Inclusion of VUSs also demonstrates mutual exclusivity(*p* = 0.04, WExT). **b** VUSs in *CDK12* and *BRCA1*. Size of circle indicates LST scores, while the *blue filling* corresponds to the proportion of signature 3-related mutations. Interestingly, 3 of 4 *BRCA1* VUSs occurred in regions enriched for loss of heterozygosity (*LOH*), and are associated with an increased likelihood of a functional deficit in HR^38^. Variants discussed in text indicated in *red*

We reasoned that if bi-allelic alterations in these genes were interacting in the same pathway (i.e., HR) and epistatic, they should be mutually exclusive of each other. The detection of mutual exclusivity depends on the incidence of mutations, and the frequency of mutations in HR-related genes is low. Hence, we focused our analysis on HBOC-type cancers with evidence of HR deficiency (i.e., high LST scores or signature 3; methods). Sixteen HR-related genes harbored ≥ 2 bi-allelic pathogenic events, and demonstrated statistically significant evidence of mutual exclusivity (*p* = 0.02; COMET test, Fig. 3a; Supplementary Fig. 4), consistent with the hypothesis that these genes may be in an epistatic interaction.

### Reclassification of variants with unknown significance

We next sought to define whether bi-allelic VUSs would also be mutually exclusive in HBOC cancers. Analysis of bi-allelic pathogenic and VUS alterations again supported mutual exclusivity in cancers with genomics features of HR deficiency (Fig. 3a). Twenty-one VUSs were found to be associated with HR deficiency, including four in *BRCA1*, two in *BRCA2*, and five in CDK12 (Fig. 3b and Supplementary Data 4). Fifteen of these were exclusive of any other bi-allelic alteration, consistent with these alterations being putatively pathogenic. Of these 15 mutations, the pathogenicity of the *BRCA1* p.G1788V mutation and the *CDK12* p.L996F mutation is supported by previous clinical pre-disposition analysis and functional studies, respectively (Fig. 3b)^23, 24^. Across all cancers, approximately 6% (*n* = 488) of cases had a bi-allelic VUSs in an HR-related gene, of which 8% had genomics features consistent with HR deficiency (*n* = 91; Supplementary Data 5).

## Discussion

Massively parallel sequencing of some cancer types (e.g., non-small-cell lung cancer and melanoma) have revealed frequent, and often hotspot, mutations in oncogenes that are therapeutically targetable. These findings have led to dramatic changes in the management of patients with these tumors. This approach, however, when applied to other cancer types has proven not as fruitful, given that the prevalence of targetable mutations in oncogenes in most other common cancer types is modest^25–27^. Most of the precision medicine endeavors in oncology carried out to date have focused on targeting somatic mutations affecting oncogenes. An alternative approach for the realization of the potentials of precision medicine, however, is to match specific chemotherapy and targeted therapy agents to the deficiencies cancer cells harbor in terms of their mechanisms of DNA repair. Here, we observed that over 5% of all cancers harbor bi-allelic pathogenic mutations in HR-related genes, and that these bi-allelic genetic alterations are often associated with genomic features of HR deficiency. Importantly, these mutations are neither restricted to *BRCA1* or *BRCA2*, nor only found in tumors traditionally associated with *BRCA1* or *BRCA2* germline mutations. In fact, here we document that these bi-allelic inactivating events occur across all cancer types, providing novel opportunities for the delivery of precision medicine approaches and expanding the population of patients who may benefit from agents targeting HR DNA repair defects.

Unlike mutations in most oncogenes or tumor suppressor genes, mutations in DNA-repair related genes provide a unique opportunity to assess for their phenotypic consequences by examining structural and mutational signatures obtained from the same sequencing data used to discover these mutations^12, 28^. This is not restricted only to HR; for example, cancers with defects in nucleotide excision repair have also been shown to display mutational signatures characteristic of deficiency in this pathway and may be exquisitely sensitive to agents that produce intra-strand crosslinks^15^. Here, we further exploited this modality of genomic data to reclassify missense somatic mutations as putatively pathogenic. There is increasing evidence, however, that the presence of a genomic signature characteristic of deficiency in a particular DNA repair pathway can also be employed for critical decisions in regards to the optimal treatment of cancer patients. For example, cancers with mismatch repair have long been known to harbor microsatellite instability (MSI) and a disproportionately high number of somatic genetic alterations. Comprehensive analysis of the TCGA has recently identified a number of tumors in traditionally non-Lynch syndromic cancers, with MMR-deficiency and MSI^14^. Intriguingly, a pan-cancer basket study of MSI tumors demonstrated that these cancers display a marked response to immunotherapy, suggesting that DNA-repair deficiencies can be targeted regardless of their site of origin^29^. Taken together, our findings emphasize the potential contribution of in depth analyses of the spectra of somatic mutations and copy number alterations, in addition to the cataloguing of the repertoire of somatic mutations in cancers, for the rendering of critical therapeutic decisions for individual cancer patients.

Our study, however, has important limitations. Although we found a correlation between bi-allelic pathogenic mutations in HR-related DNA repair genes and genomic evidence of HR deficiency (signature 3 and LST) across cancer types, the basis of HR deficiency remains unexplained in a large number of cases. It should be noted that in this study we focused on specific types of somatic and germline SNVs and indels, and did not consider somatic rearrangements affecting HR-related genes or methylation of the promoter of these genes, which may account for some of the unexplained cases^28^. Another limitation of our study stems from the fact that whole-exome sequencing data were employed to determine HR deficiency; recent whole-genome analyses have suggested that whole-exome sequencing has a lower sensitivity and specificity than whole-genome sequencing or functional assays to determine HR deficiency^20, 28^. In fact, we have recently demonstrated that in breast cancer, over 89% of cases lacking competent HR as defined by lack of RAD51 foci formation in an ex-vivo functional assay of HR deficiency harbor a bi-allelic alteration affecting an HR-related gene, and that 89% of breast cancers with bi-allelic inactivation of a HR-related gene are unable to elicit RAD51 foci formation following ex-vivo IR^20^. Importantly, however, our results warrant further studies to test whether an even stronger correlation between bi-allelic inactivation of HR-related genes and lack of competent HR DNA repair across cancer types would be observed if HR deficiency was defined by whole-genome sequencing or functional assays rather than on the basis of whole-exome sequencing data.

Despite these limitations, here we demonstrate that bi-allelic alterations in HR DNA repair-related genes occur across cancer types, are mutually exclusive, and are associated with genomics features consistent with lack of competent HR DNA repair. Although we cannot rule out other causes of HR deficiency (e.g., some dominant negative or haploinsufficient mono-allelic alterations, gene rearrangements and epigenetic effects), our observations support bi-allelic genetic alterations affecting HR-related genes as a major etiology of HR DNA repair deficiency not only in cancers linked to HBOC, but across cancer types. Finally, leveraging the mutually exclusive nature of these bi-allelic hits and their genomic effects allowed us to re-examine VUSs and provided strong circumstantial evidence to reclassify some as likely pathogenic. These findings provide new avenues for HR-directed therapies in a much broader population of cancer patients and a novel method to reclassify missense mutations in regards to their functional significance.

## Methods

### Data acquisition

Somatic mutation calls were determined from mutation annotation file (MAF) for each individual cancer downloaded from Broad firehose on 01/28/16. The TCGA cancer types in the study were adrenocortical carcinoma (ACC), bladder cancer (BLCA), breast cancer (BRCA), cervical cancer (CESC), glioblastoma (GBM), head and neck squamous cell carcinoma (HNSC), kidney chromophobe (KICH), renal clear cell carcinoma (KIRC), renal papillary cell carcinoma (KIRP), low grade glioma (LGG), hepatocellular carcinoma (LIHC), lung adenocarcinoma (LUAD), lung squamous cell carcinoma (LUSC), ovarian cancer (OV), pancreatic adenocarcinoma (PAAD), pheochromocytoma and paraganglioma (PCPG), prostate adenocarcinoma (PRAD), sarcoma (SARC), melanoma (SKCM), stomach cancer (STAD), endometrial carcinoma (UCEC), uterine carcinosarcoma (UCS), colon cancer (COAD), and rectal cancer (READ). To assess copy number changes and loss of heterozygosity (LOH) for each cancer, Affymetrix SNP Array 6.0 (SNP6) array data from TCGA was downloaded from GDAC on 1/28/15. SNP6 array data were processed together, quantile-normalized, and median-polished with Affymetrix power tools. Genotyping was performed with the birdseed algorithm. PennCNV was employed to generate log R ratio and B-allele frequencies^30^. Allele-specific copy number information was generated with ASCAT^31^. Allele-specific copy number information was used to determine loss of heterozygosity for an individual mutation.

### Mutation assessment

A list of 102 homologous recombination (HR)-related genes was manually curated by three authors (S.N.P., N.R., P.B.) using their own experience and the literature^2, 4, 5, 20, 32, 33^. We have included in the list only genes for which familial segregation and/ or data derived from functional experiments linking a gene directly or indirectly to HR DNA repair. Genes were categorized as involving core HR machinery (“core”), or involved in closely related process such as DNA damage signaling or replication-associated break repair (“related”; Supplementary Data 1).

We defined pathogenic mutations as those that would clearly have an effect on the function of a gene, namely, frameshift, nonsense, start/stop codon changes, and splice site mutations. All other types of missense mutations were considered VUS. Germline variants were detected with HaplotypeCaller from the Genome Analysis Toolkit (GATK v.3/3) in the gvcf mode with default settings^34^. For germline variants, only those present in both normal and tumor samples were retained. Somatic mutations were extracted from MAFs for each individual cancer as determined by the TCGA and classified as pathogenic following the approach employed to germline mutations (namely, frame shift, nonsense, start/stop codon changes, and splice site variants). Germline VUSs were not considered further in any subsequent analysis, given the large number of germline missense variants in HR DNA repair-related genes. Additional details on germline pipeline analysis are available at https://github.com/rj67/germVar2.

We considered bi-allelic pathogenic altered tumors to refer to cases where both alleles of an HR-related gene in the tumor were inactivated. More specifically this refers to either (i) a germline pathogenic mutation with LOH of the wild-type allele, (ii) a germline pathogenic mutation and a somatic pathogenic mutation, (iii) a somatic pathogenic mutation with LOH of the wild-type allele, or (iv) two different somatic pathogenic mutations. Tumors that had bi-allelic alterations in an HR-related gene where one of the alterations was not inactivating were considered to have bi-allelic VUS alterations. This category included (i) a pathogenic germline mutation with a somatic VUS, (ii) a somatic pathogenic mutation and a somatic VUS, and (iii) a somatic VUS with LOH of the wild-type allele. Biallelic VUSs with genomic evidence of HR deficiency (i.e., elevated LST or samples where signature 3 was dominant (see below)) and were mutually exclusive from other bi-allelic pathogenic alterations were considered putatively pathogenic.

### Genomic features of HR DNA repair deficiency

We computed both structural and mutational signatures indicative of HR deficiency. We first determined LST from allele specific segmented data following methods outlined in the initial publications^8, 9, 35^. An LST cutoff of 15 as per the initial report was used to define HR deficiency status in breast, ovarian and prostate cancers^8^. We used non-negative least squares regression to determine the proportions of mutations in each sample that were similar to the mutational signatures in cancer as described by Alexandrov et al.^12^., to determine if a mutational signature of HR deficiency was present. This process determines the proportion of mutations due to a particular mutational signature. We then determined which signature was responsible for the majority of mutations, and deemed that the dominant signature in a particular cancer. HBOCs were considered to have features of HR DNA repair if they had a high LST score or signature 3 was the dominant signature in the sample.

### Statistics

Comparisons of LST or the proportion of mutations due to signature 3 between different groups were performed with the Wilcox-rank sum test unless otherwise specified. Hereditary breast and ovarian syndromic cancers (HBOC) were considered to include breast, ovarian, and prostate cancer in all analysis. We also performed simulations (*N* = 100,000) randomly selecting 102 genes (the size of the HR gene panel) and deriving an empirical *p*-value for the association of alterations in HR genes and phenotypic genomic measures of HR deficiency in breast, prostate, and ovarian cancers. Results from these were concordant with results from the Wilcox-rank sum test (empirical *p*-value for LST: *p* = 0.002, empirical *p*-value for signature 3: *p* = 0.005). To identify if bi-allelic mutations were mutually exclusive, we first limited cases to those that are likely to have HR deficiency by restricting to tumors with elevated LST or where signature 3 was the dominant signature. We used WExT, a saddle point approximation of COMET to determine if the 16 genes with at least two bi-allelic pathogenic alterations in HBOC cancers were statistically mutually exclusive^36, 37^. We subsequently performed permutation testing by shuffling the mutation matrix with HR genes by keeping the row sums constant (i.e., mutation rate/gene remains constant) and computing a test statistic per COMET. COMET creates a multi-dimensional contingency table and constructs a test statistic to identify if the observed table is more extreme, which can be computationally intractable for lager number of genes^36^. We verified mutual exclusivity for the top 10 genes (i.e., *BRCA1, BRCA2, PALB2, BRIP1, RAD51D, ATM, CHEK2, FANCE, FANCM*, and *CDK12*) using COMET for bi-allelic pathogenic alterations without bi-allelic VUS (*p* = 0.018, COMET test). Finally, all analysis were conducted in the R statistical environment (v3.3.2, http://www.r-project.org/)

### Data availability

Data and code to reproduce key components of each figure are publically available at http://www.github.com/riazn/biallelic_hr. Somatic MAFs were obtained from Broad Institute’s firehose pipeline at https://gdac.broadinstitute.org/. SNP6 array data to determine loss-of-heterozygostiy and calculate LST were obtained from TCGA at https://portal.gdc.cancer.gov/.

## Electronic supplementary material

Supplementary Information

Supplementary Dataset 1

Supplementary Dataset 2

Supplementary Dataset 3

Supplementary Dataset 4

Supplementary Dataset 5
