# Supplementary material for: Pan-cancer analysis of bi-allelic alterations in homologous recombination DNA repair genes
Source: Nat Commun. 2017 Oct 11;8:857. doi: 10.1038/s41467-017-00921-w (PMC5636842; doi:10.1038/s41467-017-00921-w)
Supplement: Supplementary file 1 — Supplementary Information [file 41467_2017_921_MOESM1_ESM.pdf]

Type of file: pdf

Title of file for HTML: Supplementary Information

Description: Supplementary Figures

Type of file: xlsx

Title of file for HTML: Supplementary Data 1

Description: List of homologous recombination (HR) related genes

Type of file: xlsx

Title of file for HTML: Supplementary Data 2

Description: Frequency of different types of alterations in HR genes pan-cancer.

Type of file: xlsx

Title of file for HTML: Supplementary Data 3

Description: Frequency of alteration of the HR pathway in different cancers

Type of file: xlsx

Title of file for HTML: Supplementary Data 4

Description: Details of 21 variants of unknown significance (VUS) in HBOC cancers.

Type of file: xlsx

Title of file for HTML: Supplementary Data 5

Description: Details of 91 variants of unknown significance (VUS) with genomic features of HR deficiency

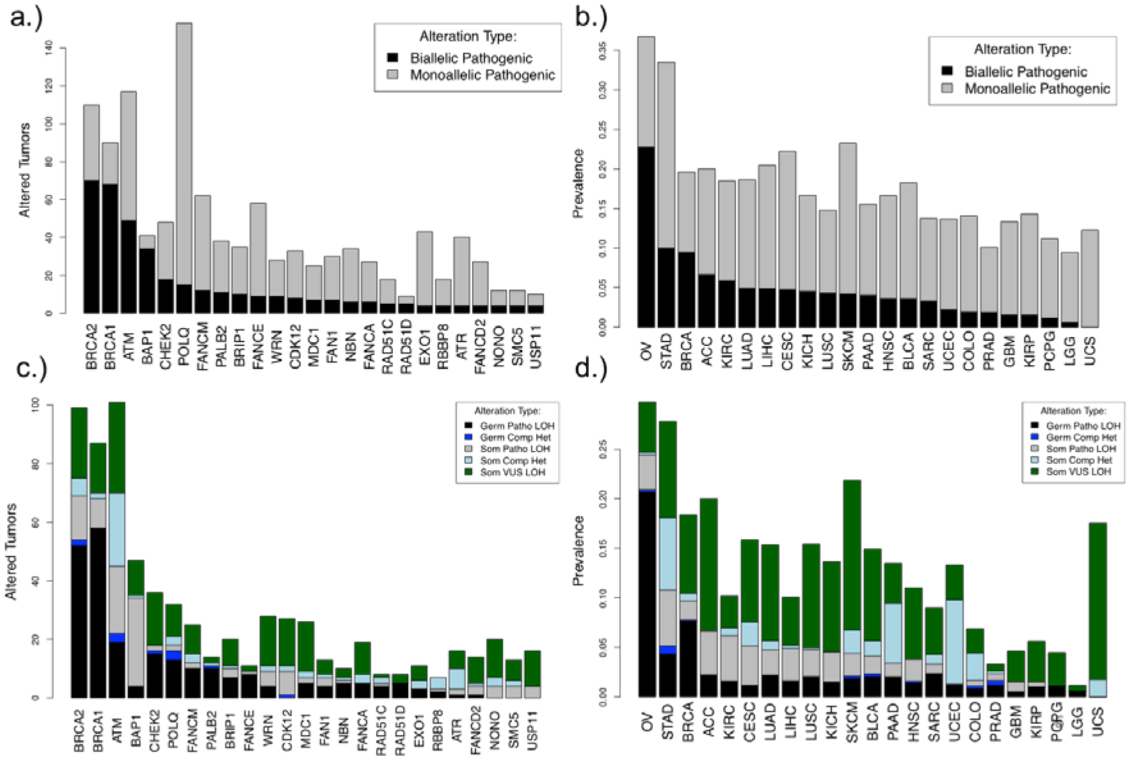

Supplementary Figure 1: Mono and bi-allelic alterations in HR-related genes across cancer types.

a.) Top 25 most frequently mutated HR-related genes stratified by bi-allelic and mono-allelic pathogenic alterations. **b.)** Frequency of bi-allelic and mono-allelic alterations in the HR-related genes stratified according to cancer type. **c.)** Characteristics and etiology of bi-allelic alterations in the top 25 most frequently mutated HR-related genes. The categories of bi-allelic alterations are: Germ Patho LOH: a pathogenic germline mutation with somatic LOH of the wild-type allele; Germ Comp Het: a germline pathogenic mutation with either a somatic pathogenic mutation or a somatic VUS; Som Patho LOH: a somatic pathogenic mutation with LOH of the wild-type allele; Som Comp Het: two somatic pathogenic mutations or one somatic pathogenic mutation and one somatic VUS; Som VUS LOH: a somatic VUS with LOH of the wild-type allele. **d.)** Characteristics and etiology of bi-allelic alterations in HR-related genes according to cancer type. Categories of bi-allelic alterations are those reported in **c**.

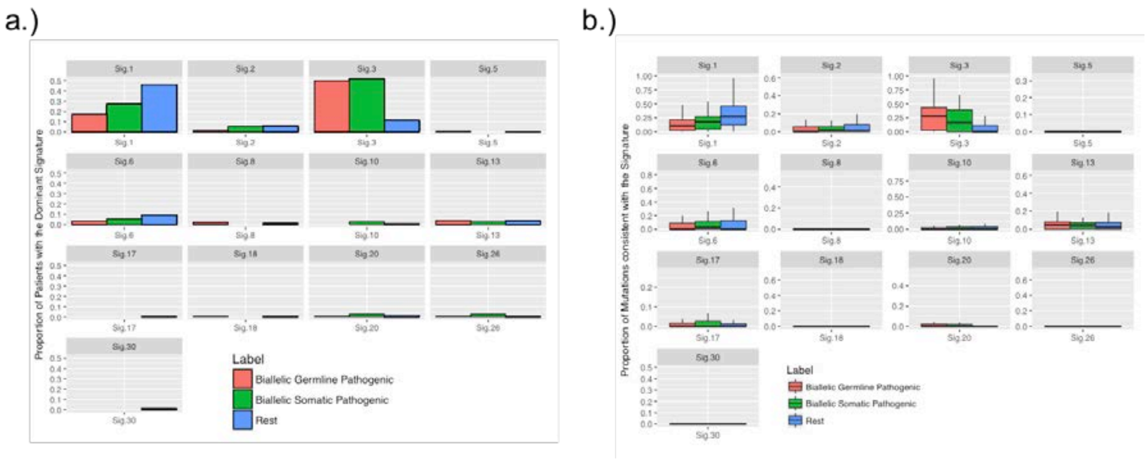

Supplementary Figure 2: Bi-allelic pathogenic alterations in HR-related genes are significantly associated with signature 3 and inversely associated with signature 1 (Signature1).

a.) Comparison of mutational signatures present in breast cancer (13 in total) analyzed in HBOCs (breast, ovarian cancer and prostate). For each tumor, the dominant signature was determined (**methods)**, and subsequently stratified by genotype to determine if an association existed. Only signature 3 (BRCA signature) was found to be directly associated with bi-allelic pathogenic alterations in HR-related genes. Of note, signature 1 (Signature1, aging signature) was found to be inversely associated with the presence of bi-allelic alterations ($p = 3.0*10^{-13}$ & $p = 0.028$; for germ-line and somatic respectively; Fisher's exact test). **b.)** Comparison of mutational signatures as in (a.), however using the proportion of mutations due to each particular signature rather than dominant signature. The relative proportion of mutations due to Signature1 was significantly lower than in the presence of bi-allelic alterations than in cancers with mono-allelic inactivation of HR-related genes and the remaining cases ($p = 2.3 * 10^{-14}$ & $p = 0.001$; for germ-line and somatic respectively; Wilcoxon rank-sum test). In both (a) & (b), the box plot center line represents the median, the box limits represent the 1st and 3rd quartiles respectively, and the whiskers extend from box limits to the largest value up to 1.5 times the interquartile range.

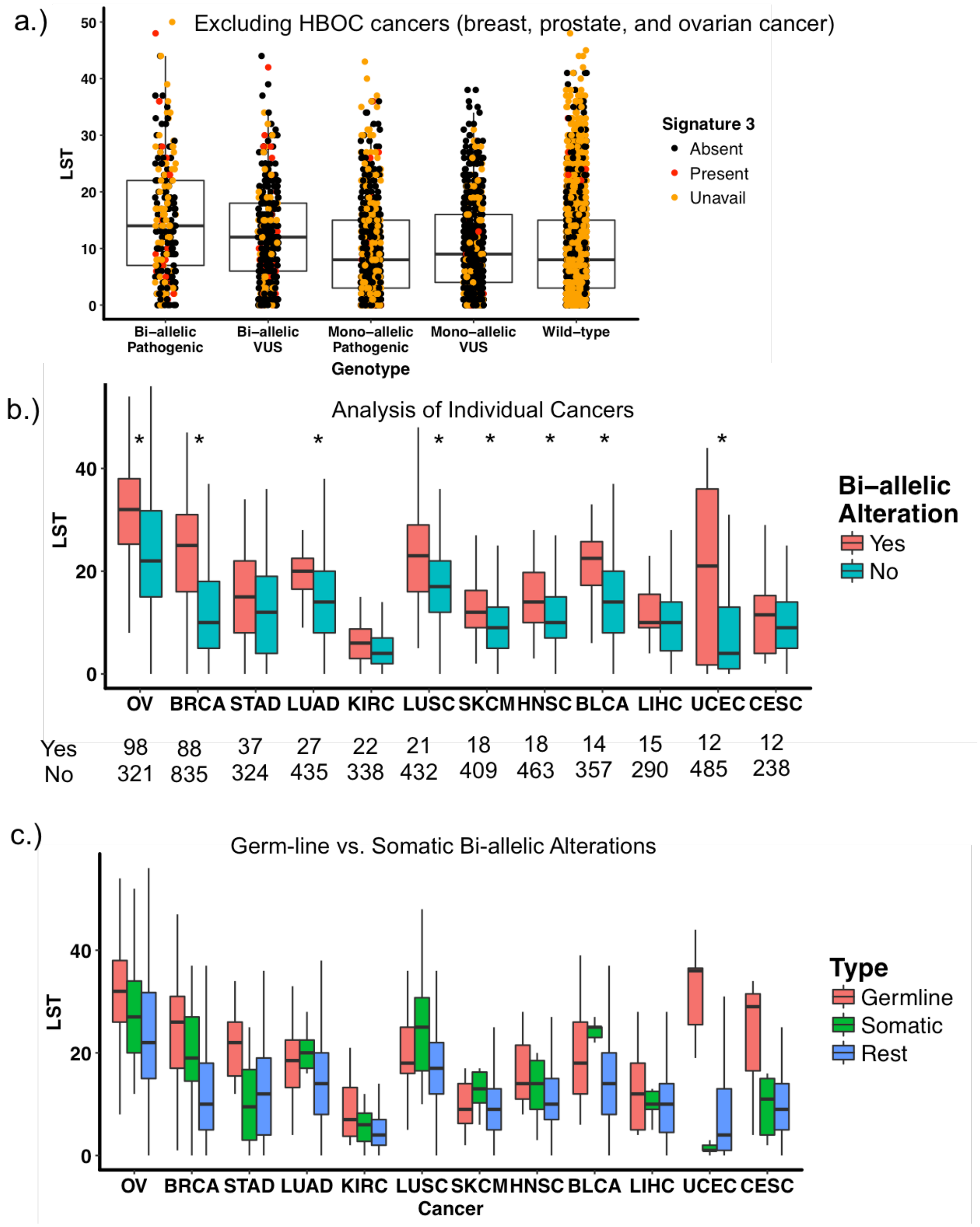

Supplementary Figure 3: Additional pan-cancer analyses

a.) Analysis excluding HBOC cancers (breast, prostate and ovarian cancer)**.** Bi-allelic pathogenic alterations in HR-related genes are significantly associated with LST and signature 3 when excluding HBOC cancers from analysis ($p = 2.79*10^{-16}$ and $p = 7.9*10^{-4}$ respectively; Wilcoxon-rank sum test). **b.)** Analysis of individual cancers with at least 10 bi-allelic alterations. There is a significant association between elevated LST and bi-

allelic alterations in 8 cancers: OV ($p = 8.9*10^{-11}$), BRCA ($p = 5.4*10^{-17}$), LUAD ($p = 1.6*10^{-3}$), LUSC (p = 0.02), SKCM (p = 0.04), HNSC (p = 0.02), BLCA ($p = 7.8*10^{-3}$), UCEC (p = 0.03); border-line in two cancers STAD (p = 0.08) and KIRC (p = 0.06); and non-significant in 2 cancers LIHC (p = 0.23) and CESC (p = 0.74). Asterisk in figure corresponds to cancers with individually significant associations. All p values were derived using the Wilcoxon-rank sum test **c.)** Influence of germ-line and somatic bi-allelic alterations in individual cancers. A multi-variate linear model controlling for cancer type demonstrated that the effect size of germ-line mutations was larger than somatic mutations for LST (germ-line coefficient = 8.2; somatic = 3.2, $p < 0.001$ for both) and for the proportion of mutations due to signature 3 (germ-line coefficient = 0.09; somatic coefficient = 0.02, $p < 0.001$ & p = 0.06 respectively). In all panels included in this Figure, the box plot center line represents the median, the box limits represent the 1st and 3rd quartiles respectively, and the whiskers extend from box limits to the largest value up to 1.5 times the interquartile range.

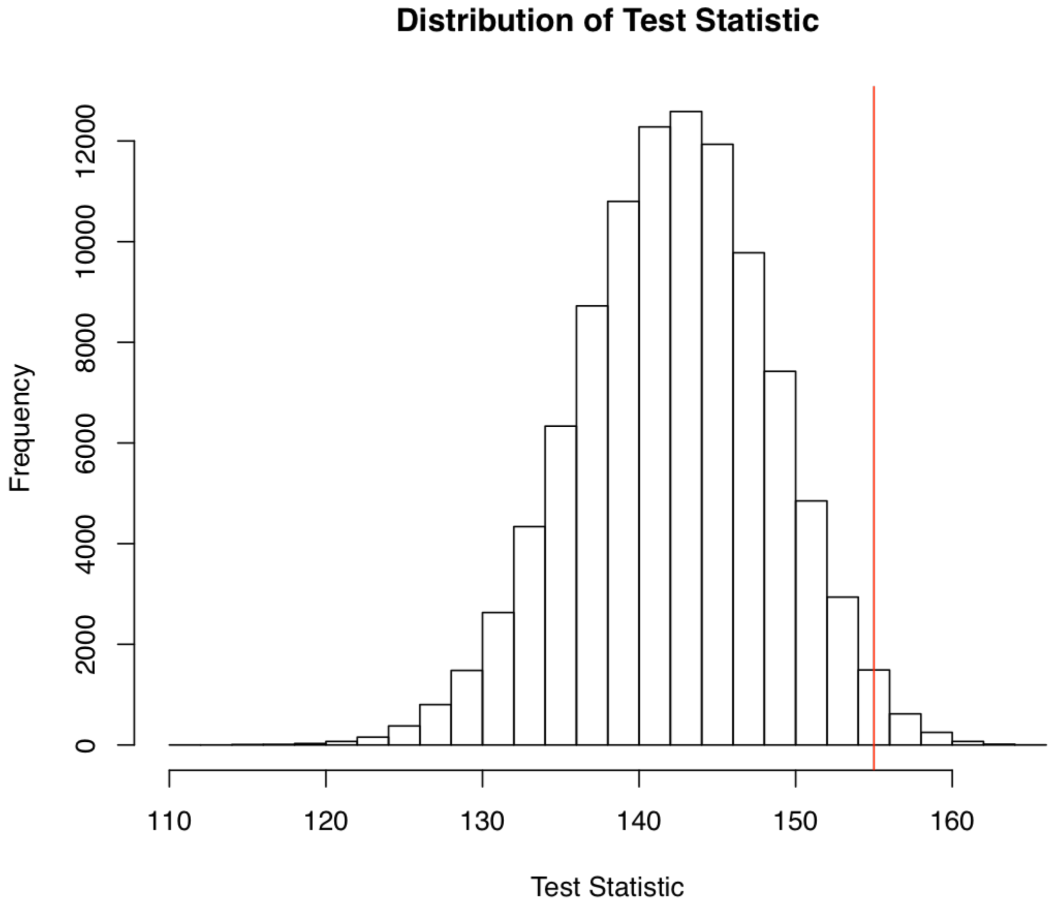

Supplementary Figure 4: Permutation testing further verifies mutual exclusivity of HR-related genes in HBOC cancers.

A permutation test (n=100,000) based on COMET confirmed the mutual exclusivity of top 16 altered HR-related genes in HBOC cancers (p=0.025) (**methods** for details).
